# A multicenter study of clinical impact of variant of uncertain significance reclassification in breast, ovarian and colorectal cancer susceptibility genes

**DOI:** 10.1002/cam4.5202

**Published:** 2022-11-24

**Authors:** Sukh Makhnoon, Brooke Levin, Megan Ensinger, Kristin Mattie, Robert J. Volk, Zhongming Zhao, Tito Mendoza, Sanjay Shete, Laila Samiian, Generosa Grana, Andrew Grainger, Banu Arun, Brian H. Shirts, Susan K. Peterson

**Affiliations:** ^1^ Department of Behavioral Science UT MD Anderson Cancer Center Houston Texas USA; ^2^ William G. Rohrer Cancer Genetics Program, Division of Hematology and Medical Oncology MD Anderson Cancer Center at Cooper University Health Care Camden New Jersey USA; ^3^ OhioHealth Cancer Genetics Program Columbus Ohio USA; ^4^ Department of Health Services Research UT MD Anderson Cancer Center Houston Texas USA; ^5^ Center for Precision Health, School of Biomedical Informatics The University of Texas Health Science Center at Houston Houston Texas USA; ^6^ Department of Symptoms research UT MD Anderson Cancer Center Houston Texas USA; ^7^ Division of Cancer Prevention and Population Sciences UT MD Anderson Cancer Center Houston Texas USA; ^8^ Baptist MD Anderson Jacksonville Florida USA; ^9^ Clinical Cancer Genetics UT MD Anderson Cancer Center Houston Texas USA; ^10^ University of Washington Seattle WA USA

**Keywords:** cancer, clinical management, hereditary, variant of uncertain significance, variant reclassification

## Abstract

**Background:**

Clinical interpretation of genetic test results is complicated by variants of uncertain significance (VUS) that have an unknown impact on health but can be clarified through reclassification. There is little empirical evidence regarding VUS reclassification in oncology care settings, including the prevalence and outcomes of reclassification, and racial/ethnic differences.

**Methods:**

This was a retrospective analysis of persons with and without a personal history of cancer carrying VUS (with or without an accompanying pathogenic or likely pathogenic [P/LP] variant) in breast, ovarian, and colorectal cancer predisposition genes seen at four cancer care settings (in Texas, Florida, Ohio, and New Jersey) between 2013 and 2019.

**Results:**

In 2715 individuals included in the study, 3261 VUS and 313 P/LP variants were reported; 8.1% of all individuals with VUS experienced reclassifications and rates varied significantly among cancer care settings from 4.81% to 20.19% (overall *p* < 0.001). Compared to their prevalence in the overall sample, reclassification rates for Black individuals were higher (13.6% vs. 19.0%), whereas the rates for Asian individuals were lower (6.3% vs. 3.5%) and rates for White and Hispanic individuals were proportional. Two‐year prevalence of VUS reclassification remained steady between 2014 and 2019. Overall, 11.3% of all reclassified VUS resulted in clinically actionable findings and 4.6% subsequently changed individuals' clinical managements.

**Conclusions:**

The findings from this large multisite study suggest that VUS reclassification alters clinical management, has implications for precision cancer prevention, and highlights the need for implementing practices and solutions for efficiently returning reinterpreted genetic test results.

## Graphical abstract

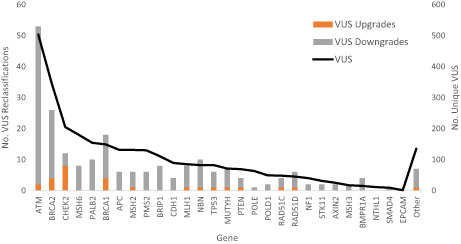

## 1 INTRODUCTION

Clinical interpretation of genetic test results is increasingly complicated by variants of uncertain significance (VUS)^1^ that have an unknown impact on health. Over half of all interpreted variants are VUS^2^ with reported frequencies in large cohorts ranging from 10% to 41% when testing is done using large multigene panels.^3^, ^4^, ^5^, ^6^ Reclassification can clarify a variant's clinical significance and is increasingly facilitated by the availability of updated information about normal human genomic diversity, especially among underrepresented minority populations. Reclassification of genetic variants is now a routine occurrence in genomic medicine as the field accumulates epidemiological, clinical, and functional evidence to enable variant reinterpretation. While all types of variants have the potential to undergo reclassification, VUS must be reinterpreted to be clinically meaningful. Patients whose test results indicate a VUS are typically not advised to alter risk management or clinical care based on their genetic test result, and the potential for reclassification is routinely discussed during genetic counseling. Although reclassified VUS are prevalent and have the potential to change clinical management, relatively little is known about the clinical experiences of returning reclassified VUS results in oncology and their subsequent impact on patients' care management.

Variants can undergo reclassification either over time as enough people carrying that specific variant undergoes genetic testing or through purposeful variant reclassification efforts. The majority of VUS are reclassified or downgraded, to benign or likely benign variants (B/LB), while a smaller proportion is upgraded to pathogenic or likely pathogenic variants (P/LP) that can change clinical management. VUS reclassification rates can vary based on a number of factors including patient characteristics such as ancestry^7^ and differences in variant reinterpretation policies of genetic testing laboratories.^8^ Number of family members with phenotypic disease,^9^ and year of initial genetic testing (pre or post‐2015 American College of Medical Genomics guideline issuance^1^) have also been reported as additional factors associated with higher reclassification rates.

Recontacting patients with reclassified variants is a process challenged by logistical issues (e.g., when and who should initiate reinterpretation, length of time from initial genetic testing to reclassification) as well as ethical issues (e.g., duty to reinterpret, recontact, extent and duration of these duties) that have been debated extensively.^10^, ^11^ Real‐world data on clinical experiences of VUS reclassification may help facilitate a consensus on these contentious issues. Clinical oncology also faces unique challenges around variant reclassification as patients may undergo genetic testing in a setting other than where they receive cancer care. In such cases, providers who ordered the genetic testing rather than oncology care providers would be notified of variant reclassifications, thus any new finding may not be included in clinical decision‐making regarding cancer care or management. Tertiary cancer hospitals in particular treat patients from all over the world, often without coordinated care between oncologists and primary care providers, which presents a challenge for returning reclassified results that often take years to be interpreted.

There is insufficient empirical evidence regarding VUS reclassification in oncology care settings, including the prevalence and outcomes of reclassification, racial and ethnic differences, and the proportion of patients who change their medical management as a result of VUS reclassification. Such data are critical to developing evidence‐based guidelines and standards for returning reclassified results. The existing literature on variant reclassification report reclassification experiences of major commercial laboratories, and the evidence from clinical settings is limited by their small sample sizes, inclusion of data from single institutions, and a lack of focus on VUS reclassification. To address this gap, we examined the prevalence and clinical impact of VUS reclassification across several years in four geographically dispersed cancer care settings.

## 2 METHODS

### 2.1 Study population

Patients who underwent genetic counseling at four U.S. cancer care settings during specified time periods were included in this study. Sites were MD Anderson Cancer Center in Houston, Texas (2013–2019), and three member institutions from the MD Anderson Cancer Network (https://www.mdanderson.org/about‐md‐anderson/our‐locations/md‐anderson‐cancer‐network.html) including Cooper University Health Care in Camden, New Jersey (2013–2018), Baptist Health System in Jacksonville, Florida (2014–2018), and OhioHealth in Columbus, Ohio (2014–2018). These four geographically dispersed institutions represent unique health care systems and differ in many ways from MD Anderson as well as from each other.^12^ MD Anderson is a comprehensive cancer center with a patient population from across the United States and the world, the three network sites provide cancer care as a component of their health care system and serve patients from their city and region. Importantly, all sites employ cancer genetic counselors who ensure the availability of on‐site genetic counseling services per the Commission on Cancer accreditation requirement. Overall, genetic counseling practices (e.g., EMR documentation, referral, scheduling, genetic testing) are comparable across sites with some individual level practice variations with regard to gene panels ordered, laboratory used for testing.

This study included all adult individuals (age 18 years or older) who had a VUS (with or without an accompanying P/LP variant) in a breast, ovarian or colorectal cancer susceptibility gene. Patients were first identified using prospective clinical databases maintained at the four study sites that included individuals who underwent testing for a single gene, multiple genes, or both (if they had more that one of these tests). A retrospective medical record review was performed for all patients with a waiver of written informed consent and approval from the institutional review boards of all four participating institutions (PA19‐0403). Data collection time periods varied across intuitions based on the availability of prospective clinical databases that enabled the identification of patients who had undergone genetic counseling and was limited to 2019 or earlier to allow sufficient time for variant reclassification to occur.

### 2.2 Data collection

Demographic (race/ethnicity, sex, age) and clinical (cancer status, date of cancer diagnosis) data were extracted from the clinical databases and electronic medical records (EMRs). In order to focus on hereditary breast, ovarian, and colorectal cancers, we considered VUS detected in the following breast‐ ovarian‐, and colorectal‐cancer‐associated genes: *APC, ATM, AXIN2, BRCA1, BRCA2, BMPR1A, BRIP1, CHEK2, CDH1, EPCAM, MLH1, MSH2, MSH3, MSH6, MUTYH, NBN, NF1, NTHL1, POLD1, POLE, PALB2, PMS2, PTEN, RAD51C, RAD51D, SMAD4, STK11*, and *TP53*. Patients who only had a P/LP variant, without an accompanying VUS were excluded from this analysis. For patients who received an amended report, details associated with variant reclassification (upgrades and downgrades), date of initial genetic testing, and length of time for reclassification were recorded. Data on clinical management following variant reclassification associated with the gene of interest were also collected including surgical decisions and cancer surveillance. Charts were reviewed between January and August 2021.

### 2.3 Data analysis

We summarized demographic (age, sex, self‐reported race/ethnicity), clinical characteristics (personal history of cancer) as mean (SD) or number (percentage). T*ime to VUS reclassification was defined as the interval from the date of initial genetic testing to the date of issue of an amended genetic test report. For each year of initial genetic testing, we calculated the proportion of VUS reclassified within following 2‐year time period as we had at least 2 years of follow‐up on all individuals, We performed Chi‐square test for differences in proportions*. The statistical significance level was set at α = 0.05. We used R software (Version 3.4.4) for all statistical analyses.

## 3 RESULTS

A total of 3301 patients with non‐negative genetic test results in hereditary breast, ovarian or colorectal cancer genes were identified from the study sites. Ninety‐nine patients were not included in the analysis as they did not have a VUS in one of the defined cancer susceptibility genes. An additional 645 patients were excluded as they only had a P/LP variant, and 40 patients had inaccessible medical records, leaving 2715 patients in the final analytic sample. Table 1 shows the sociodemographic and clinical characteristics of patients included in the study. Of all patients, 11.0% (300/2715) had a P/LP variant in addition to a VUS. A total of 3261 VUS and 313 P/LP variants were reported across all individuals (Table S1).

**TABLE 1:** Characteristics individuals with variant of uncertain significance (VUS) across three to four study sites (*N* = 2715)

Variable	Category	Cooper	MD Anderson	OhioHealth	Baptist	Total
		*n* = 515 (%)	*n* = 1685 (%)	*n* = 358 (%)	*n* = 157 (%)	*N* = 2715 (%)
Date of genetic counseling		2013–2018	2013–2019	2014–2018	2014–2018	2013–2019
Sex	Female	497 (96.50)	1392 (82.61)	337 (94.13)	147 (93.63)	2373 (87.40)
	Male	36 (6.99)	293 (17.39)	21 (5.87)	10 (6.37)	360 (13.26)
Race/Ethnicity	NH Asian	17 (3.30)	138 (8.19)	13 (3.63)	3 (1.91)	171 (6.30)
	Hispanic or Latino	44 (8.54)	252 (14.96)	4 (1.12)	0 (0.00)	300 (11.05)
	NH Black	90 (17.48)	206 (12.23)	42 (11.73)	31 (19.75)	369 (13.59)
	NH White	349 (67.77)	1039 (61.66)	291 (81.28)	118 (75.16)	1797 (66.19)
	Other	11 (2.14)	17 (1.01)	2 (0.56)	5 (3.18)	35 (1.29)
	Unknown	4 (0.78)	33 (1.96)	6 (1.68)	0 (0.00)	43 (1.58)
Personal history of cancer	Breast	270 (52.43)	820 (48.66)	260 (72.63)	80 (50.96)	1350 (49.72)
	Ovarian	15 (2.91)	123 (7.30)	24 (6.70)	19 (12.1)	162 (5.97)
	Colorectal	9 (1.75)	138 (8.19)	21 (5.87)	6 (3.82)	168 (6.19)
	Other	94 (18.25)	525 (31.16)	31 (8.66)	8 (5.1)	650 (23.94)
	Unaffected	127 (24.66)	79 (4.69)	22 (6.15)	44 (28.03)	228 (8.40)
Genetic testing year	2002–2009	2 (0.39)	12 (0.71)	0 (0.00)	0 (0.00)	14 (0.52)
	2010–2012	0 (0.00)	12 (0.71)	0 (0.00)	1 (0.64)	13 (0.48)
	2013–2015	93 (18.06)	273 (16.20)	44 (12.29)	4 (2.55)	414 (15.25)
	2016–2018	401 (77.86)	919 (54.54)	304 (84.92)	151 (96.18)	1775 (65.38)
	2019–2020	18 (3.50)	464 (27.54)	10 (2.79)	1 (0.64)	493 (18.16)
Test result type	VUS only	458 (88.93)	1493 (88.61)	320 (89.39)	144 (91.72)	2415 (88.95)
	VUS + P/LP	57 (11.07)	192 (11.39)	38 (10.61)	13 (8.28)	300 (11.05)
Variant reclassification		104 (20.19)	81 (4.81)	27 (7.54)	14 (8.92)	226 (8.32)

Of the 2715 patients with VUS results, 8.1% (220/2715) experienced reclassifications including both upgrades and downgrades. As shown in Table 2, of 3261 unique variants initially classified as uncertain, 7.36% (240/3261) were reclassified: 88.7% were downgraded from VUS to B/LB, whereas 11.3% were upgraded to P/LP variants. P/LP variants accompanying VUS were reclassified less commonly (2.23%) but most reclassifications were upgrades from LP to P. Some variants were reclassified more than once and many patients experienced multiple variant reclassifications. Variant reclassification rates varied significantly among study sites with the lowest rate of 4.81% reported at MD Anderson, with higher but comparable rates of 7.54% and 8.92% at OhioHealth and Baptist, and the highest rate of 20.19% reported at Cooper (overall *p* < 0.001). Of note, the two sites with the higher reclassification rates also had a slightly higher proportion of Black individuals and nearly a quarter had no personal history of cancer.

**TABLE 2:** Variant reclassifications by type

	No. Unique Variants (*n* = 3574)		
	P/LP	VUS	Total
No. Initially detected	313	3261	3574
No. Reclassified (%)^a^	7 (2.23)	240 (7.36)	247 (6.91)
Upgraded (%)	5 (71.4)	27 (11.3)	32 (12.9)
Downgraded (%)	2 (28.6)	213 (88.7)	215 (87.1)

^a^ Some underwent both upgrade and downgrade; Variant reclassifications were defined as downgrades if the variant was reclassified from P/LP to VUS, P to LP, or from VUS to B/LB, and upgrades if the convserse happened.

**FIGURE 1:**
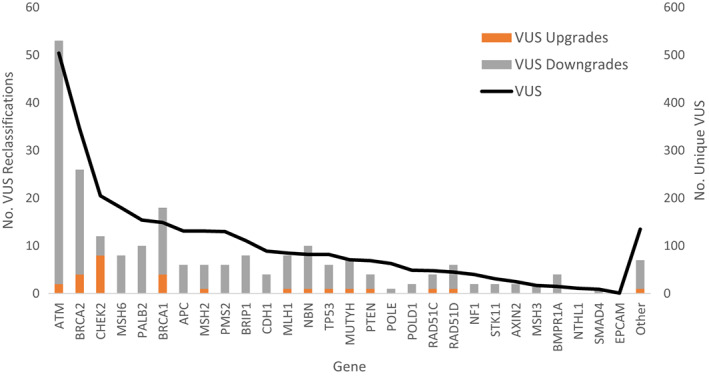
Number of reclassified unique variants of uncertain significance (VUS) by gene as a function of the total number of VUS. The number of unique VUS is shown on the right axis and that number of reclassifications are on the left axis.

**FIGURE 2:**
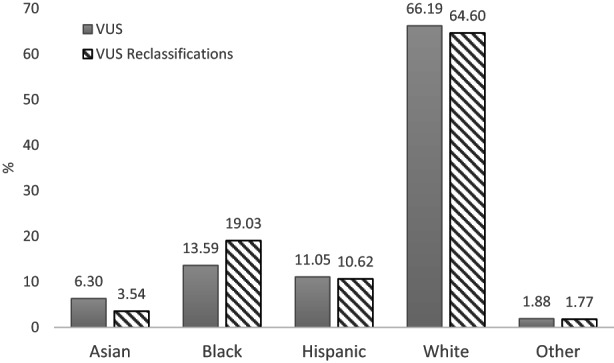
No of patients with VUS reclassifications by race/ethnicity compared to their number in the total sample.

Figure 1 shows the VUS reclassification pattern across genes and suggests that VUS in most genes underwent reclassification at a rate proportional to their prevalence in the overall sample with few exceptions (e.g., *ATM* and *BRCA1*). VUS reclassifications occurred most in *ATM, BRCA2, BRCA1*, and *CHEK2* with only VUS downgrades reported in some genes. Figure 2 shows VUS reclassification rates by patient race/ethnicity and illustrates that VUS reclassification rates for Blacks were higher compared to their prevalence in the sample (19.0% vs. 13.6%), whereas the rates for Asians were lower (3.5% vs. 6.3%) and reclassification rates for Whites and Hispanics where proportional to their prevalence in the overall sample.

**FIGURE 3:**
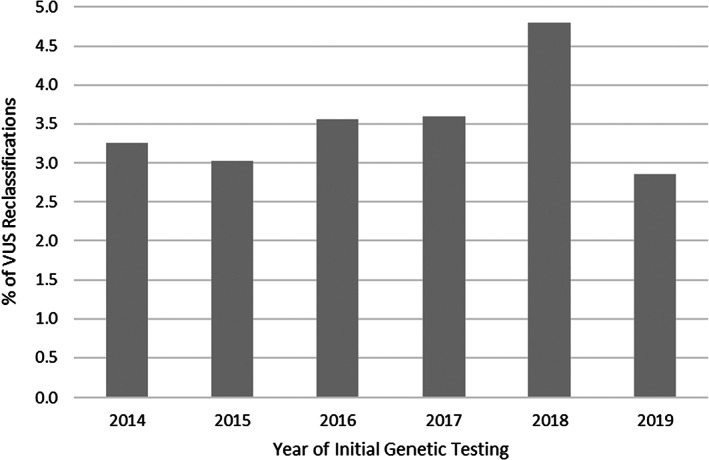
Year‐specific 2‐year prevalence of variant of uncertain significance reclassification.

Figure 3 shows the 2‐year prevalence of VUS reclassification as a function of the year the initial VUS was reported. Between 2014 and 2019, proportion of VUS reclassified within 2 years of reporting remained steady overall with approximately 3–4.5% of VUS reclassifications per 2‐year period. Unadjusted median time to VUS reclassification decreased steadily between 2014 and 2019 from 3.08 years to 0.91 years (data not shown).

Overall, 11.3% of all reclassified VUS were clinically actionable and 4.6% subsequently changed patients' clinical managements. In total, 27 VUS were upgraded to P/LP variants in one of the following genes: *ATM, BARD1, BRCA1, BRCA2, CHEK2, MLH1, MSH2, MUTYH, NBN, PTEN, RAD51C, RAD51D*, and *TP53* (Table 3). For 12 patients, medical management recommendations were altered in response to VUS reclassification by starting more intensive screening regimens (mammography, breast MRI, colonoscopy, etc.) or undergoing risk‐reducing surgeries including bilateral mastectomy or bilateral salpingo‐oophorectomy. Of the remaining 15 patients, eight had previously undertaken the risk management as indicated by VUS reclassification, two patients insufficient follow‐up time after reclassification to assess any management change, two patients passed away before variant reclassification, and clinical data were not available for three patients.

**TABLE 3:** Details of clinically actionable VUS reclassifications and resulting management change

Patient No	Variant reclassification	Management change	Sex	Race	Cancer Diagnosis
Patient 1	*CHEK2* (VUS to LP)	Yes, began high risk screening using breast MRI	F	White	Unaffected
Patient 2	*BRCA2* (VUS to LP)	Yes, underwent BLM and RRSO	F	White	Breast
Patient 3	*BRCA2* (VUS to LP)	Yes, began mammography	F	White	Breast
Patient 4	*CHEK2* (VUS to LP)	Yes, began high risk screening for breast and ovarian cancers	F	White	Breast
Patient 5	*CHEK2* (VUS to LP)	Yes, began frequent colonoscopy per NCCN guidelines for CHEK2	F	White	Breast
Patient 6	*BRCA1* (VUS to LP)	Yes, BLM in past; RRSO not covered by insurance; OC screening being performed now	F	Hispanic or Latino	Breast
Patient 7	*PTEN* (VUS to P)	Yes, undergoing breast MRI post reclass; Not interested in prophylactic mastectomy	F	Hispanic or Latino	Colon
Patient 8	*CHEK2* (VUS to P)	Yes, began breast MRI; Already had lumpectomy, Not interested in prophylactic mastectomy	F	White	Breast
Patient 9	*BRCA1* (VUS to LP)	Yes, underwent BLM after VUS upgrade	F	White	Breast
Patient 10	*CHEK2* (VUS to LP)	Yes, more frequent breast cancer screening	F	White	Breast
Patient 11	*ATM* (VUS to LP)	Yes, underwent BLM	F	White	Breast
Patient 12	*BRCA1* (VUS to LP)	Yes, began frequent mammography	F	Black	Breast
Patient 13	*RAD51D* (VUS to P)	No, previously underwent TAH/BSO for fallopian tube cancer	F	White	Fallopian tube
Patient 14	*CHEK2* (VUS to LP)	No, previously underwent left mastectomy due to cancer	M	White	Breast
Patient 15	*BRCA2* (VUS to LP)	No. Already post BLM and BSO as VUS classified as LP at a different lab	F	White	None
Patient 16	*CHEK2* (VUS to LP)	No. Mastectomy previously due to cancer and undergoing annual mammography (no MRI) at time of upgrade	F	White	Breast
Patient 17	*TP53* (VUS to LP)	No, previously underwent BLM; part of screening study for other cancers from before reclassification	F	White	Leukemia
Patient 18	*BRCA2* (VUS to LP)	No, previously underwent BSO; not interested in BLM; breast MRI planned but none performed yet	F	Black	Unaffected
Patient 19	*MLH1* (VUS to LP)	No, already underwent TAHBSO	F	Other	Breast
Patient 20	*MUTYH* (VUS to LP)	No, undergoing colonoscopies every 5 years at time of upgrade	M	White	Unaffected
Patient 21	*ATM* (VUS to LP)	No, previously underwent BLM and TLH BSO	F	White	Breast
Patient 22	*MSH2* (VUS to LP)	No, surgical colectomy in past; plans for more frequent colonoscopy but not enough time since reclassification	M	White	Sarcoma
Patient 23	*RAD51C* (VUS to LP)	NA, already underwent TAHBSO due to OC; deceased before upgrade	F	White	Ovary
Patient 24	*NBN* (VUS to P)	NA, Patient deceased before upgrade	M	White	Pancreas
Patient 25	*CHEK2* (VUS to LP)	Unknown, no follow‐up data after reclassification	F	Hispanic or Latino	Breast
Patient 26	*BARD1* (VUS to LP)	Unknown, no follow‐up data after reclassification	F	White	Unknown
Patient 27	*TP53* (VUS to LP)	Unknown, no follow‐up data after reclassification	M	Other	Unaffected

Abbreviations: BC, breast cancer; BLM, bilateral mastectomy; MRI, magnetic resonance imaging; OC, ovarian cancer; TAHBSO, total abdominal hysterectomy bilateral salpingo oophorectomy; VUS, variant of uncertain significance; Sites 1, 2, 3, and 4 refer to Baptist, Cooper, MD Anderson, and OhioHealth respectively.

Experience with re‐contacting patients upon variant reclassification is difficult to systematically ascertain using EMR data as not all experiences of returning results are documented, however genetic counseling summary notes offer some insight into the process. Anecdotally, two patients were informed of their reclassifications three or more years after variant reclassification as the laboratory had sent the amended report to the ordering provider who had never informed the patient of these reclassifications. For patients who died before reclassification or stopped getting care at the hospital, either their family members were informed, or a note was left in their EMR.

## 4 DISCUSSION

In this multisite study of 2715 patients with VUS in breast, ovarian, and colorectal cancer susceptibility genes, reclassification changed the clinical management for at least 0.4% of all patients. Of all the reclassified VUS, the majority were downgraded, however, 11.3% were upgraded and thus clinically actionable. A subset of patients with upgraded variants had previously undertaken risk management recommendations associated with the change in variant reclassification, and some were unevaluable, however, 4.6% of reclassified VUS resulted in altered clinical management for persons with and without a personal history of cancer. This demonstrates that it is not uncommon for VUS reclassification to alter the clinical management and highlights the need for standardized clinical practice guidelines and policies for returning reclassified VUS results to patients.

Overall variant and VUS reclassification rates reported in this study were 6.91% and 7.36% respectively. A range of VUS reclassification rates have been previously reported in literature which have ranged between 8 and 28% in reports from clinical cancer care settings,^7^, ^9^, ^13^, ^14^, ^15^ and anywhere between 6.9 and 66% in studies that report results from purposeful variant reclassification efforts,^16^ and 24.9% in one report from a commercial laboratory.^17^ Our results are consistent with results from other hospitals and genetic testing laboratories. Our study has the advantage of including patients from multiple cancer care sites across four U.S. states, and inclusion of a highly actionable set of cancer susceptibility genes compared to prior publications that often only include broad spectrums of less well‐understood genes from single cancer care settings.

We report significant variation in VUS reclassification rates among the four cancer care settings (from 4.8% to 20.2%) which is a novel addition to the literature. Although these reclassification rates are within the ranges previously reported in literature,^7^, ^13^, ^14^, ^15^ the underlying reason for this variation remains unclear. We believe, the most plausible explanation is the variation in racial/ethnic characteristics of patients seen across the four sites. It is unlikely that personal history of cancer contributes to VUS reclassification as all individuals have enough family history of cancer to trigger genetic testing and thus variant interpretation, however, unaffected individuals may be younger and have more dynamic family history which may affect reclassification. Eligible genes for inclusion in this study were limited to those associated with breast, ovarian, and colorectal cancer susceptibility, however, less well‐understood genes that are more likely to undergo reclassification were included in some genetic testing panels. As shown in Figure 1, these other genes were a very small proportion of the overall sample and likely had limited impact on the observed variation in reclassification rates across sites. Although there was some variation in years of initial genetic testing across sites, most testing occurred after issuance of 2015 ACMG guidelines for variant reclassification. All study sites used a common set of commercial genetic testing laboratories and none used in‐house academic laboratory. Although data on specific laboratories were not assessed in this study, it could have contributed to the variation in reclassification across sites as they are known to have different variant reclassification policies.^8^

Racial differences in VUS reclassification have been reported previously with markedly higher with reclassification rates in Asians (38–45%),^18^, ^19^, ^20^ and some indication of variation in reclassification rates by ancestry.^7^ Although, our study confirms reports of variation in VUS reclassification rates by ancestry, we find that compared to the prevalence of VUS in the overall sample, VUS reclassification rates are lower among Asians, higher among Blacks, and proportional among NH Whites and Hispanics. Although the exact reason for the racial/ethnic differences is unclear, it may be explained in part by the availability of updated information about normal human genomic diversity, especially among underrepresented minority populations. Specific variant level exploration to understand the evidence base that resulted in these reclassifications is warranted. An ongoing concern in cancer genetics is the disproportionally high VUS rates among racial minorities^21^ combined with limited confidence that oncologists have with interpreting and correctly managing patients with VUS.^22^, ^23^ VUS reclassification is a clear way to alleviate this concern and the encouraging rate of VUS reclassification for Black individuals in this study suggests progress. Going forward, it will be important to address the continued underrepresentation of racial minorities in genomic research to solve the VUS problem as broader genetic testing becomes commonplace in oncology practice.

Two‐year prevalence of VUS reclassification in this study remained stable between 2014 and 2019. Unadjusted time to reclassification decreased over the years to 0.91 years for a VUS initially detected in 2019 which is similar to times previously reported in literature^15^, ^17^ but only reflects the inherent right censored nature of the data. In contrast to previous publications with considerable length‐biased data on time‐to‐reclassification, we report a 2‐year prevalence of VUS reclassification to more accurately estimate the right‐censored time to VUS reclassification. Studies with longer follow‐up time from contemporary clinical cohorts are needed to better understand the burden of VUS reclassification that clinicians face.

Anecdotal reports of recontacting patients with reclassified VUS from this study not only exemplify longstanding ethical and logistical issues around variant and VUS reclassification^10^ but they also represent unique challenges that cancer care settings face around this increasingly common phenomena. The economic considerations are particularly salient for patient management as reinterpretation may result in dramatic impacts on healthcare utilization patterns, quality of life of index patients as well as their relatives. Payment and reimbursement policies for care resulting from reclassified variants remain underdeveloped and there is a need for specific guidance for clinical providers. Insofar as clinical actionability, preventability, treatability, and severity of disease motivate efforts to recontact patients with reinterpreted variants, the genes investigated in this study represent those with compelling indications for recontacting. Yet, lapses in timely recontact occurred which underscores the need for implementing practices and solutions for efficiently returning reinterpreted genetic test results.

Secondary cancer prevention through screening and early detection including mammography, breast MRI, and colonoscopy as well as prophylactic surgeries such as mastectomy were observed in among those with VUS upgrades in this study. This demonstrates the clinical utility of VUS reclassification for individuals both with and without a personal history of cancer. Reclassification from P, LP, or VUS to B or LB could also have important consequences for patient management—for example, discontinuation of intensive screening regimens provided to people at high risk of cancer. Patient with VUS who adopted prevention behaviors before reclassification were motivated by their personal history of cancer (patient 16, 19, 21,) significant family history of cancer (patient 20), participation in screening research study (patient 17), or because they received conflicting interpretation of variant from different laboratories (patient 15). We observed no instance of LP variant being downgraded to VUS/LB/B in this study and LP variants upgraded to P did not require change in clinical management.

This study has several strengths, including a large multi‐institutional sample from geographically dispersed cancer care settings and inclusion of VUS from all major breast, ovarian, and colorectal cancer susceptibility genes which represents the genes with highest clinically utility. However, results from this study should be considered in light of several limitations including the oversimplified categorization of race in EMRs and thereby our study. More appropriate subcategorizations of race (e.g., Asians) were not possible in this study due to the source data limitations but should be explored in future work. Findings from four cancer care settings show variation due to demographic and clinical characteristics which may not be generalizable to other populations.

### 4.1 Conclusion

In summary, clinically relevant VUS reclassification is not a rare event in oncology. Hereditary cancer is currently the most common application for genetic testing and the high rate of VUS combined with desire for accurate VUS reclassification and the significant management change that it can result in highlights the magnitude of the issue in clinical oncology. Variation in reclassification rates across cancer care settings, racial differences in VUS reclassification experiences, and anecdotal delays in recontact upon VUS reclassification highlights the need for solutions around VUS reclassification and recontact.

## AUTHOR CONTRIBUTIONS

The authors confirm their contribution to the paper as follows: study conception and design: Makhnoon S, Shete S, Peterson SK; data collection: Makhnoon S, Levin B, Ensinger M, Mattie K, Arun B, Grainger A, Laila S, Grana G; analysis and interpretation of results: Makhnoon S, Shete S, Shirts BH, Peterson S; draft manuscript preparation: Makhnoon S, Peterson SK; supervision: Volk B, Mendoza T, Zhao Z, Peterson SK, Shete S. All authors reviewed the results and approved the final version of the manuscript.

## FUNDING INFORMATION

SM's work was supported by a training grant from the National Cancer Institute Career Development Award (K99CA256216), HERA Ovarian Cancer Foundation Award (FP00008751), and Cancer Prevention and Research Institute of Texas – CPRIT (Award# RP170259). ZZ was partially supported by the National Institutes of Health grant (R01LM012806) and the Cancer Prevention and Research Institute of Texas grants (CPRIT RP180734 and RP210045). RJV is supported by the Hubert L. and Olive Stringer Distinguished Professorship in Cancer Research.

## CONFLICT OF INTEREST

The authors have no relevant financial or non‐financial interests to disclose.

## ETHICS APPROVAL

The study was approved by the Institutional Review Boards of UT MD Anderson Cancer Center, Cooper University Health Care, Baptist, and OhioHealth. This research comprised retrospective collection of data obtained for clinical purposes.

## Supporting information


Figure S1

## Data Availability

The data generated in this study are available upon request from the corresponding author.

## References

[1] Richards S , Aziz N , Bale S , et al. Standards and guidelines for the interpretation of sequence variants: a joint consensus recommendation of the American College of Medical Genetics and Genomics and the Association for Molecular Pathology. Genet Med. 2015;17:405‐424. 25741868 10.1038/gim.2015.30 PMC4544753

[2] Starita LM , Ahituv N , Dunham MJ , et al. Variant interpretation: functional assays to the rescue. Am J Hum Genet. 2017;101:315‐325. 28886340 10.1016/j.ajhg.2017.07.014 PMC5590843

[3] K. A. Idos GE , Ricker C , Sturgeon D , et al. Multicenter prospective cohort study of the diagnostic yield and patient experience of multiplex gene panel testing for hereditary cancer risk. JCO Precis Oncol. 2019;3:1‐12. 10.1200/PO.18.00217 PMC8260917 34322651

[4] Lincoln SE , Yang S , Cline MS , et al. Consistency of BRCA1 and BRCA2 variant classifications among clinical diagnostic laboratories. JCO Precis Oncol. 2017;1:1‐10. 10.1200/PO.16.00020 PMC5542009 28782058

[5] Frey MK , Kim SH , Bassett RY , et al. Rescreening for genetic mutations using multi‐gene panel testing in patients who previously underwent non‐informative genetic screening. Gynecol Oncol. 2015;139:211‐215. 26296696 10.1016/j.ygyno.2015.08.006

[6] Shirts BH , Casadei S , Jacobson AL , et al. Improving performance of multigene panels for genomic analysis of cancer predisposition. Genet Med. 2016;18:974‐981. 26845104 10.1038/gim.2015.212

[7] Slavin TP , van Tongeren LR , Behrendt CE , et al. Prospective study of cancer genetic variants: variation in rate of reclassification by ancestry. J Natl Cancer Inst. 2018;110:1059‐1066. 29618041 10.1093/jnci/djy027 PMC6249694

[8] Garrett LT , Hickman N , Jacobson A , et al. Family studies for classification of variants of uncertain classification: current laboratory clinical practice and a new web‐based educational tool. J Genet Couns. 2016;25:1146‐1156. 27422780 10.1007/s10897-016-9993-2 PMC5114323

[9] Wright M , Menon V , Taylor L , Shashidharan M , Westercamp T , Ternent CA . Factors predicting reclassification of variants of unknown significance. Am J Surg. 2018;216:1148‐1154. 30217367 10.1016/j.amjsurg.2018.08.008

[10] Appelbaum PS , Parens E , Berger SM , Chung WK , Burke W . Is there a duty to reinterpret genetic data? The Ethical Dimensions Genet Med. 2020;22:633‐639. 31616070 10.1038/s41436-019-0679-7 PMC7185819

[11] El Mecky J , Johansson L , Plantinga M , et al. Reinterpretation, reclassification, and its downstream effects: challenges for clinical laboratory geneticists. BMC Med Genomics. 2019;12:170. 31779608 10.1186/s12920-019-0612-6 PMC6883538

[12] Bednar EM , Walsh MT , Baker E , et al. Creation and implementation of an environmental scan to assess cancer genetics Services at Three Oncology Care Settings. J Genet Couns. 2018;27:1482‐1496. 10.1007/s10897-018-0262-4 PMC6240000 29770910

[13] Turner SA , Rao SK , Morgan RH , Vnencak‐Jones CL , Wiesner GL . The impact of variant classification on the clinical management of hereditary cancer syndromes. Genet Med. 2019;21:426‐430. 29875428 10.1038/s41436-018-0063-z

[14] Macklin S , Durand N , Atwal P , Hines S . Observed frequency and challenges of variant reclassification in a hereditary cancer clinic. Genet Med. 2018;20:346‐350. 29215655 10.1038/gim.2017.207

[15] Chiang J , Chia TH , Yuen J , et al. Impact of variant reclassification in cancer predisposition genes on clinical care. JCO Precis Oncol. 2021;577‐584:577‐584. 10.1200/PO.20.00399 34994607

[16] Tsai GJ , Rañola JMO , Smith C , et al. Outcomes of 92 patient‐driven family studies for reclassification of variants of uncertain significance. Genet Med. 2019;21:1435‐1442. 30374176 10.1038/s41436-018-0335-7

[17] Mersch J , Brown N , Pirzadeh‐Miller S , et al. Prevalence of variant reclassification following hereditary cancer genetic testing. Jama. 2018;320:1266‐1274. 30264118 10.1001/jama.2018.13152 PMC6233618

[18] So MK , Jeong TD , Lim W , et al. Reinterpretation of BRCA1 and BRCA2 variants of uncertain significance in patients with hereditary breast/ovarian cancer using the ACMG/AMP 2015 guidelines. Breast Cancer. 2019;26:510‐519. 30725392 10.1007/s12282-019-00951-w

[19] Ha HI , Ryu JS , Shim H , Kong SY , Lim MC . Reclassification of BRCA1 and BRCA2 variants found in ovarian epithelial, fallopian tube, and primary peritoneal cancers. J Gynecol Oncol. 2020;31:e83. 33078592 10.3802/jgo.2020.31.e83 PMC7593220

[20] Lee JS , Oh S , Park SK , et al. Reclassification of BRCA1 and BRCA2 variants of uncertain significance: a multifactorial analysis of multicentre prospective cohort. J Med Genet. 2018;55:794‐802. 30415210 10.1136/jmedgenet-2018-105565

[21] Kurian AW , Ward KC , Abrahamse P , et al. Time trends in receipt of germline genetic testing and results for women diagnosed with breast cancer or ovarian cancer, 2012‐2019. J Clin Oncol. 2021;39:1631‐1640. 33560870 10.1200/JCO.20.02785 PMC8274804

[22] Macklin SK , Jackson JL , Atwal PS , Hines SL . Physician interpretation of variants of uncertain significance. Fam Cancer. 2019;18:121‐126. 29721668 10.1007/s10689-018-0086-2

[23] Kurian AW , Li Y , Hamilton AS , et al. Gaps in incorporating germline genetic testing into treatment decision‐making for early‐stage breast cancer. J Clin Oncol. 2017;35:2232‐2239. 28402748 10.1200/JCO.2016.71.6480 PMC5501363

