# Supplementary material for: A multicenter study of clinical impact of variant of uncertain significance reclassification in breast, ovarian and colorectal cancer susceptibility genes
Source: Cancer Med. 2022 Nov 24;12(3):2875–84. doi: 10.1002/cam4.5202 (PMC9939195; doi:10.1002/cam4.5202)
Supplement: Supplementary file 1 — Figure S1 [file CAM4-12-2875-s001.docx]

**SUPPLEMENTARY MATERIALS**

**Figure S1:** Types of variants reported across genes.

P/LP: pathogenic or likely pathogenic; VUS: variant of uncertain significance
